# Using the Quantum Potential in Elementary Portfolio Management: Some Initial Ideas

**DOI:** 10.3390/e23020180

**Published:** 2021-01-30

**Authors:** Hossein Khaksar, Emmanuel Haven, Sina Nasiri, Gholamreza Jafari

**Affiliations:** 1 Physics Department, Shahid Beheshti University, Tehran 1983969411, Iran; hossein.khaksar@hotmail.com (H.K.); gjafari@gmail.com (G.J.); 2 Faculty of Business Administration, Memorial University of Newfoundland, St John’s, NL A1C 5S7, Canada; 3 Department of Banking and Finance, Eastern Mediterranean University, Famagusta 99628, North Cyprus, Turkey; nasiri@iasbs.ac.ir

**Keywords:** finance, portfolio and risk management, quantum physics

## Abstract

Owing to the globalization of the economy, the concept of entangled markets started to form, and this occurrence has smoothed the entrance of quantum mechanics into behavioral finance. In this manuscript, we introduce quantum risk and perform an analysis on portfolio optimization by controlling the quantum potential. We apply this method to eight major indices and construct a portfolio with a minimum quantum risk. The results show quantum risk has a power law behavior with a time-scale just as a standard deviation with different exponents.

## 1. Introduction

The growth of the global economy has made home bound investors become, almost by default, international investors. Clearly, portfolio theory plays a key role in international investments. Much research has focussed on finding the optimal strategy for allocating wealth among various assets in such a manner to reduce risk rather than maximize returns. If we adopt a hedging perspective, we can claim this is the main objective of portfolio theory [1]. Harry Markowitz’s mean-variance model [2,3] has become a gold standard in portfolio theory. The mean-variance portfolio optimization model is highly dependent on the estimation errors of sample moments and includes negative weights for large portfolios, which requires investors to take on short positions. In the case where short positions are prohibited, constraints have to be applied on portfolio weights in the optimization process [4,5,6]. A substantial amount of research has been performed so as to develop a reduced error Markowitz model. In [7,8,9,10,11,12] we observe that the various authors used different covariant matrix estimators in their models in order to achieve more accurate and diversified portfolios with a lower proportion of negative weights, especially for short time horizons (see [13,14]). Some researchers tried non-equal weighted historical data to distinguish between normal and more risky days in their portfolios [15]. Many researchers have tried to solve the problem by combining the investment horizon with return and risk. Bolgorian et al. [16] found a method to introduce a portfolio with minimized waiting time, for a particular return and known risk. In all of these methods, variance plays an important role as a classical correlation function in the process of optimization.

There is a widely held consensus in the academic community that the historical return probability density function (PDF) is in general non-Gaussian and therefore higher moments can be informative. Employing higher moments of a PDF into the estimation of risk would require hard work and in some cases might not even be possible.

However, one can attempt to change perspectives and try to apply a non-classical approach through finding the optimum solution for the portfolio problem. Although on prima facie, it may appear to be far-fetched, but the formalism of quantum mechanics can be a perfect candidate for such a situation, where the PDF is taken as an input of the theory and it gets rid of all the classical problems, including moments. It was the pioneering work of Andrei Khrennikov [17,18] which established the quantum mechanical approach in finance. Through the works of Segal [19] and Bagarello [20,21] and Haven et al. [22], amongst others, the usefulness of quantum mechanics in their applications to finance was better understood. It was Choustova [23] who first proposed to further analyze financial behavior using Bohmian quantum mechanics. Tahmasebi [24] and Shen et al. [25] used Choustova’s idea to show that historical (public) information of an asset could be stored in a quantum potential governing that asset (within a particular period of time). Nasiri et al. [26,27] used empirical methods also proposed by Tahmasebi and Shen et al. [25] to analyze the role of trading volume in the quantum potential.

In the next section, we introduce our model and formulate the questions which we shall attempt to answer in section three. There we use a genetic algorithm to optimize the introduced model in order to find the portfolio and the appropriate weights for the minimum risk. In Section 4 we compare two types of risk and we conclude in section five.

## 2. The Quantum Potential and a First Look at What We Call ‘Quantum Risk’

The concept of ‘quantum potential’ is well known as being a core part of the edifice of Bohmian mechanics [28] which is also known as the semi-classical approach to quantum mechanics. In this approach, the quantum potential plays a key role in guiding the particle to its possible trajectories. Of course, no unique particle trajectory exists in quantum mechanics. Introducing the concept of quantum potential into an interdisciplinary context can lead to ambiguity and requires an innovative interpretation. The quantum potential is easily derived from the Schrödinger PDE through the substitution of the wave function ψ in its polar form, $Re^{iS}$. Essentially, one substitutes first the polar form in the first partial time derivative of ψ (the left hand side of the Schrödinger PDE). Then, one proceeds with the polar form substitution into the second partial position derivative of ψ, which together with the multiplication of the real potential and the wave function makes up the right hand side of the Schrödinger PDE. Finally, multiplying with $e^{-iS}$ one can separate real and imaginary parts.

After the separation of real and imaginary parts, the real part equation will be derived as Equation (1), where the last term in Equation (1) is defined as the quantum potential (i.e., Q(q))

(1) $$\frac{\partial S}{\partial t}+{\left(\frac{\partial S}{\partial q}\right)}^{2}+U\left(q\right)-\frac{\hbar^{2}}{2mR}\frac{\partial^{2}R}{\partial q^{2}}=0,\;\;\;\;\;\;\;\;\;\;\;\;\;Q\left(q\right)=-\frac{\hbar^{2}}{2mR}\frac{\partial^{2}R}{\partial q^{2}}.$$

We note that U(q) is the real potential function. Tahmasebi [24] and Shen [25] showed that there exist quantum potential walls (as well as real potential walls) for an arbitrary (commodity) price return history. Nasiri [26] and Shen [25] showed that as market risk increases, the distance between the potential walls also increases.

**Figure 1:**
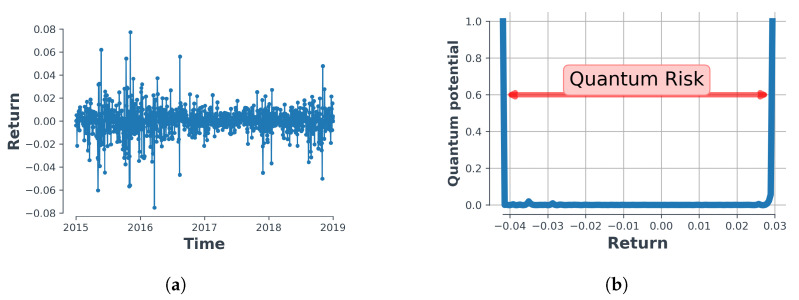
A schematic process of quantum potential and ‘quantum risk’, where (**a**) Log-return time-series plotted for S&P 500 and (**b**) quantum potential corresponding to S&P 500 time-series.

In this paper, we take the width of the potential walls as an effective measure for introducing the concept of risk. The potential walls in our method, as in Figure 1b, are defined as the walls in which the value of quantum potential becomes greater than two orders more than the mean value. The probability of finding the observed variable beyond this region becomes negligible. In order to reach a better understanding, we have illustrated the process in Figure 1, in which Figure 1b depicts the quantum potential governing an arbitrary observed time-series in a particular period of time shown in Figure 1a. The width of the walls shown in Figure 1b provides for our innovative notion which we term ‘quantum risk’ in this particular approach and we use this specific notion throughout the paper. We note that the quantum potential is effectively an infinite square well. We juxtapose standard and quantum risk in the before last section of this paper.

## 3. Portfolio Optimization

Whilst constructing a desired portfolio, it is reasonable to question why one portfolio may be preferred over another. In this paper, we use the notion of quantum risk, which we heuristically introduced in Figure 1b, in order to optimize the quantum risk associated to a portfolio. This portfolio is constructed by appropriate company shares. The return of the portfolio, or index, is defined in a straightforward way as Equation (2):

(2) $$\bar{r}\left(t\right)=\sum_{i=1}^{N}\omega_{i}r_{i}\left(t\right),$$

where $\omega_{i}$ is the weight and $r_{i}\left(t\right)=log\left(P_{i}\left(t+1\right)-log\left(P_{i}\left(t\right)\right)\right)$ is the log-return and $P_{i}\left(t\right)$ is the price of the ith security at time *t*. One can easily construct the quantum potential $\frac{-\hbar^{2}}{2mR}\frac{\partial^{2}R}{\partial q^{2}}$ where $R(\bar{r})$ is the probability density function of the portfolio return index. The measurable risk to be minimized is given by the width of the potential’s walls.

**Figure 2:**
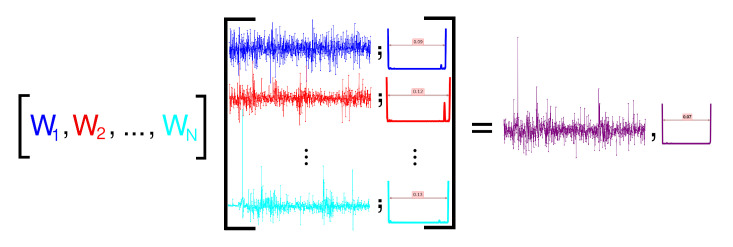
Schematic portfolio selection with quantum potential.

Our proposed method consists in finding a suitable choice of $\omega_{i}^{}$’s with the help of a genetic algorithm in order to minimize the portfolio Q-risk which we shall define in Section 4 below. A graphical illustration of the process is shown in Figure 2 in which an arbitrary set of weights is considered to construct the portfolio index.

Each of these signals has been specified with their appropriate quantum potential and their wall width, as our new ‘measure’ of quantum risk. We note that the value of 0.09 minimizes quantum risk.

One can follow through the optimization stages defined below in order to follow the process in a comprehensive manner.

I Data input:
  In the process of optimization, the first step is to collect each component of the portfolio time series data and enter them into the program as input. The markets used in the following manuscript as input of the algorithm are Dow Jones industrial, S&P 500 composite, FTSE 100, TOPIX, DAX 30 performance, NIKKEI 225, Korea SE composite and the Shanghai SE A Share. Each of the aforementioned indices can be traded within stock exchange markets and hence, they can form any arbitrary portfolio.
II Constructing weight population:
  After the given set of market series, we need an individual set of weights to initialize portfolio construction as indicated in Equation (2). In the genetic algorithm, a group of randomly generated individuals, called population, will be generated. The populations as well as the set of markets series will be the input of the evolving genetic algorithm. The Pseudo-code of the process is presented in the Appendix A.
III Optimization of population:
  Once we have the initial random populations to start with, we can evolve the population using genetic algorithm techniques and generate new populations with lower risks. The Pseudo-code of the optimization process using genetic algorithm as well as the flowchart of the process is provided for you at the end of the paper.
  In the above Pseudo-code, data is a list composed of all the markets time-series in which, data[i] would be the time-series of i’th market. At each step of the optimization process, the minimum fitness would be saved in order to be an indication of that step. The next paragraph will depict the results of optimization process discussed in here.

In what follows, we demonstrate some simulation studies and illustrate the optimum values for the weights of the indices composing the appropriate portfolio. A very noticeable fact about an optimal portfolio is that it reduces the risk of losing money. But not all the feasible combinations of securities promise to do so. By the method introduced in the previous section, we are going to apply portfolio management to the top major market indices (i.e., the Dow Jones industrial, S&P 500 composite, FTSE 100, TOPIX, DAX 30 performance, NIKKEI 225, Korea SE composite and the Shanghai SE A Share). The scaled quantum risk of these indices is shown in Figure 4b compared with the quantum risk of one arbitrarily optimized portfolio. Some of the combinations (of indices) will show the lower risk amongst all portfolios. We note the scaled quantum risk is defined as the risk which governs the scaled return of each market.

**Figure 3:**
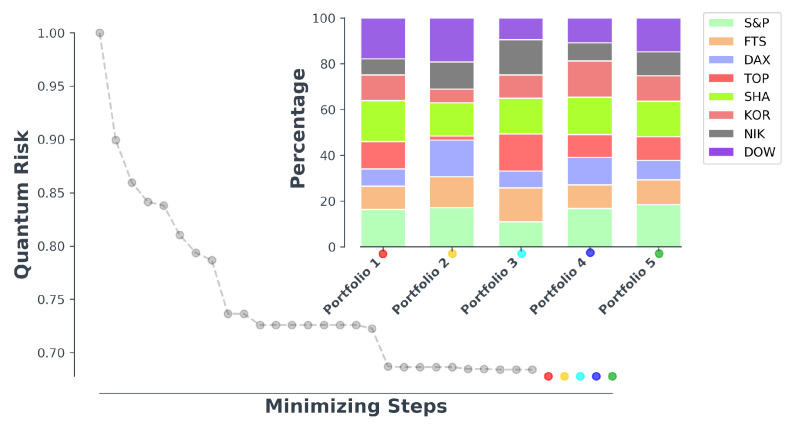
Portfolio optimization process and selected optimized portfolios.

In this paper, we have used the genetic algorithm to find the suitable combinations which minimize the quantum risk. It is quite clear that the desired condition is not satisfied with only one solution. However, multiple answers may give rise to the minimum quantum risk. Five different combinations of indices (forming 5 different portfolios) with minimum desired quantum risk for each portfolio is shown in Figure 3. We note we can expect such levels of low risk given we work with portfolios of indices.

## 4. Comparing Standard and Quantum Risk

Considering the price of the portfolio at time *t* on a daily scale, P(t), then the return price with lag τ is defined by:

(3) $$r_{\tau }\left(t\right)=\log\left(P\left(t+\tau \right)\right)-\log\left(P\left(t\right)\right).$$

The standard deviation of the return-price on the scale of τ is then $\sigma \left(\tau \right)=\sqrt{\langle r_{\tau }{\left(t\right)}^{2}\rangle -{\langle r_{\tau }\left(t\right)\rangle }^{2}}$ and Q-risk also is a function of the τ–scale. We calculate Q-risk for various time scales τ for optimized portfolio $\left\{r_{\tau }\left(t\right)\right\}$. Our results show that there exists a power-law relation between the Q-risk, and τ as follows:

(4) $$Q-\mathrm{risk}\left(\tau \right)\propto \tau^{\alpha },$$

where Q-risk(τ) is the risk of the scaled log-return of the original series for τ days. We define Q-risk as being proportional to the distance between two walls of the square well potential. We note that the walls of the potential emerge from the tails of the probability density function associated with the return-price, when very high return-prices occur with very low probability. The quantum potential as formulated in Equation (1), shows how the walls occur when we divide by such low probabilities. We believe the Q-risk definition can be justified because the larger the width between walls, the larger the variance of the return-price will be and therefore uncertainty/risk is increased. In other words, the closer we are to the walls of the potential, the more standard deviations we are removed from the mean of the probability density function. Since we can calculate the return-price (Equation (3)) with a variable lag scale (τ), we obtain a return-price of a portfolio which is function of τ. The Q-risk is thus also a function of the τ–scale. Variance is a scaling parameter and risk being proportional to the variance, has a scaling behavior (power-law) too. Hence, the smaller or longer the time scales, the smaller or the larger the fluctuations will be. We expect that Q-risk has the same behavior as our analysis of the real data shows.

**Figure 4:**
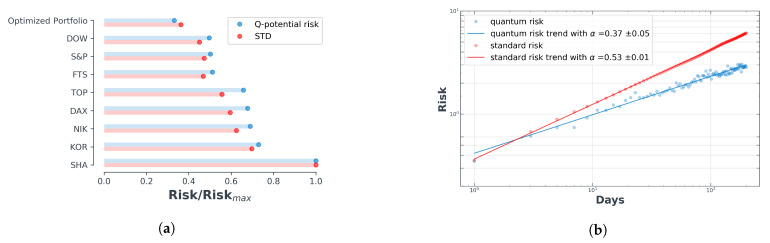
Log-return time-series and its appropriate quantum risk (Q-risk) plotted for S&P 500 index. (**a**) Comparison of standard risk with quantum risk for various countries’ stock market indices and optimized portfolio; (**b**) Quantum risk shows a scaling behavior same as the standard risk with a lower exponent.

One can examine the α exponent for different portfolios and make a comparison between their exponents. In Figure 4b, we have illustrated the amount of α for one of the selected portfolios in Figure 3, where quantum risk and standard risk plays a role for Risk. We make two observations: (i) the standard risk and quantum risk have the same trend (i.e., upward); (ii) an α close to 0.5 indicates random walk.

In Figure 4b, we have demonstrated the scaled risk for different portfolios, in order that one can get a better feeling towards comparing the risk of each individual index with its standard deviation and also the optimized portfolio, for a determined period of time (from December 1994 to December 2019). One can readily follow from Figure 4b that quantum risk has a power-law behavior which is the same as a standard risk with lower exponent. Power-law behavior shows how the risk is propagated in the scale. By knowing the risk on one scale we know it in the various other scales. The exponent of quantum risk is lower than standard risk. If we know the risk in for example a one-day scale, we expect the lower risk at a larger scale than standard risk. We emphasize that the sources of risk in standard and quantum risk are different. One source depends on the variance, but the source of quantum risk comes from the tails of the PDF tails (involving higher moments). For Gaussian PDFs, the variance is a suitable parameter but in non-Gaussian PDFs, the second moment, does not store all the available information and higher moments are thus needed in order to verify the existing information. Financial series are known as being non-Gaussian PDFs. In addition, important events are located within the tail region of the pdf, and the quantum potential exhibits available information living in those regions. Figure 4a shows the normalized quantum risk of major indices introduced in the previous section, compared with their normalized standard notion for risk which is standard deviation. As one can follow from Figure 4a, the Dow Jones and S&P 500 have the lowest risk amongst all the indices. The Shanghai index itself got the highest risk among these 8 indices. The top bars in Figure 4a show the risk and standard deviation of one selected optimized portfolio. It is clear that both the quantum risk and standard deviation of the selected portfolio is less than all the indices composing the portfolio.

## 5. Conclusions

In this paper, we showed how a simple concept of the quantum mechanical formalism can begin to aid us in risk management. We introduced a method which, if we control the quantum potential, results in extracting a specific measure of risk information of an index. Since the quantum potential has been shown to help in analyzing the coupling between markets, the bridge between market risk and systematic risk may be worthy of further consideration in future work.

## Figures and Tables

**Figure A1:**
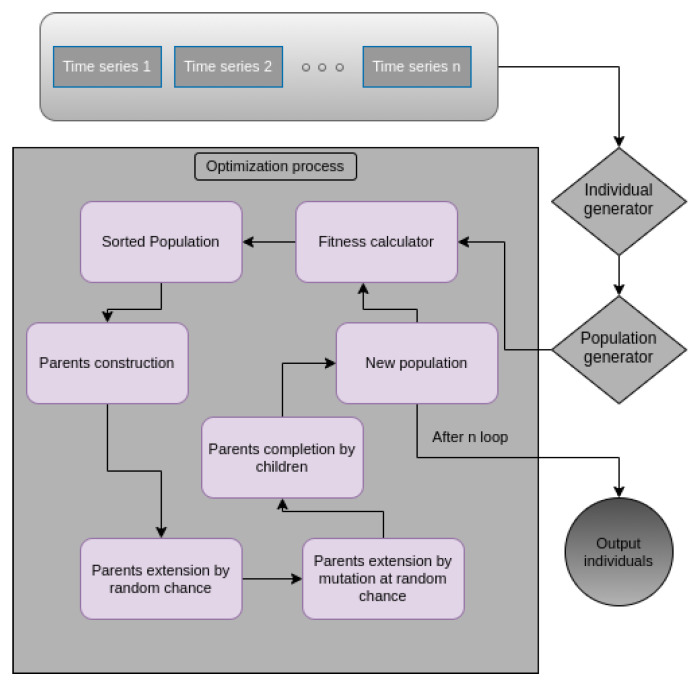
The flowchart of the portfolio optimization algorithm.

## Data Availability

The data presented in this study are openly available in: Finance.yahoo.com.
